# Identification of a Histidine Metal Ligand in the *argE*-Encoded *N*-Acetyl-L-Ornithine Deacetylase from *Escherichia coli*

**DOI:** 10.1186/2193-1801-2-482

**Published:** 2013-09-23

**Authors:** Wade C McGregor, Danuta M Gillner, Sabina I Swierczek, Dali Liu, Richard C Holz

**Affiliations:** Contribution from the Department of Chemistry, Marquette University, Milwaukee, WI 53233 USA; Chemistry and Biochemistry, Loyola University Chicago, Chicago, IL 60626 USA; The Department of Chemistry, Silesian University of Technology, Gliwice, 44-100 Poland; The Department of Applied Sciences and Mathematics, College of Technology and Innovation, Arizona State University, Mesa, AZ 85212 USA

**Keywords:** Zinc, Bioinorganic Chemistry, Hydrolysis, Deacetylase, Active site ligands, Isothermal titration calorimetry, Mechanistic enzymology

## Abstract

The H355A, H355K, H80A, and H80K mutant enzymes of the *argE*-encoded *N*-acetyl-L-ornithine deacetylase (ArgE) from *Escherichia coli* were prepared, however, only the H355A enzyme was found to be soluble. Kinetic analysis of the Co(II)-loaded H355A exhibited activity levels that were 380-fold less than Co(II)-loaded WT ArgE. Electronic absorption spectra of Co(II)-loaded H355A-ArgE indicate that the bound Co(II) ion resides in a distorted, five-coordinate environment and Isothermal Titration Calorimetry (ITC) data for Zn(II) binding to the H355A enzyme provided a dissociation constant (*K*_d_) of 39 μM. A three-dimensional homology model of ArgE was generated using the X-ray crystal structure of the *dapE*-encoded *N*-succinyl-L,L-diaminopimelic acid desuccinylase (DapE) from *Haemophilus influenzae* confirming the assignment of H355 as well as H80 as active site ligands.

Prokaryotes synthesize arginine through a series of eight enzymatically-catalyzed reactions that differ from those of eukaryotes by two key steps: i) acetylation of glutamate and ii) the subsequent deacetylation of the arginine precursor *N*^α^-acetyl-L-ornithine (L-NAO) by the *argE*-encoded *N*^α^-acetyl-L-ornithine deacetylase (ArgE) (Figure 1) (Cunin et al. 1986; Davis 1986; Ledwidge and Blanchard 1999). The arginine biosynthetic pathway is found in all Gram-negative and most Gram-positive bacteria including all of the so-called ESKAPE pathogens (***E****nterococcus faecium,****S****taphylococcus aureus,****K****lebsiella pneumoniae,****A****cinetobacter baumannii,****P****seudomonas aeruginosa,* and ***E****nterobacter* species), which account for more than 60% of the multidrug resistant hospital acquired infections in the United States (Velasco et al. 2002; Cunin et al. 1986; Sakanian et al. 1990; Xu et al. 2000; Sakanyan et al. 1996; Hani et al. 1999; Valentsev et al. 1995; Picard and Dillon 1989)*.* Because ornithine is required, not only for the synthesis of arginine in bacteria, but also for the production of polyamines involved in DNA replication and cell division, the L-NAO deacetylation step is critical for bacterial proliferation (Girodeau et al. 1986). Indeed, when an arginine auxotrophic bacterial strain void of L-NAO deacetylase activity was transformed with a plasmid containing *argE*, an Arg^+^ phenotype resulted (Meinnel et al. 1992). However, when the start codon (ATG) of *argE* in the same plasmid was changed to the Amber codon (TAG), the resultant plasmid was unable to relieve arginine auxotrophy in the same cell strain indicating that ArgE is required for cell viability. Given the fact that ArgE is only found in prokaryotes and is required for bacterial cell growth and proliferation, it represents a potential enzymatic target for the development of a new class of antimicrobial agents (Girodeau et al. 1986).

**Figure 1:**
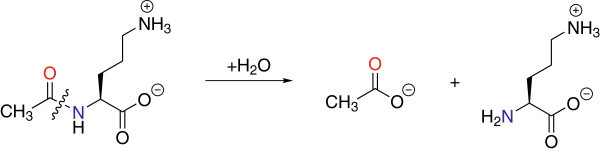
**Reaction catalyzed by ArgE.**

At the present time, no X-ray crystallographic data is available for any ArgE enzyme but it shares sequence homology with several dinuclear Zn(II) metallopeptidases in the M28 family and biochemical studies revealed that the ArgE from *E. coli* is a Zn(II) containing enzyme (Javid-Majd and Blanchard 2000; McGregor et al. 2005; Holz 2002; Holz et al. 2003). Based on sequence alignments of the ArgE from *E. coli* with the aminopeptidase from *Aeromonas proteolytica* (AAP) (Desmarais et al. 2006), the *dapE*-encoded *N*-succinyl-L,L-diaminopimelic acid desuccinylase (DapE) (Nocek et al. 2010), the *N*-acetyl-L-citrulline deacetylase (ACD) from *Xanthomonas campestris* (Shi et al. 2007), and the carboxypeptidase from *Pseudomonas sp* strain-RS-16 (CPG2) (Rowsell et al. 1997), the residues that function as ligands in the dinuclear active site of AAP, DapE, ACD, and CPG_2_ are strictly conserved in ArgE (Figure 2) (Born et al. 1998; Chevrier et al. 1994; Rowsell et al. 1997). Since AAP, DapE, and CPG_2_ possess a (μ-aquo)(μ-carboxylato)dizinc(II) core with one terminal carboxylate and one histidine residue at each metal site, a similar active site was proposed for ArgE (Tao et al. 2012).

**Figure 2:**
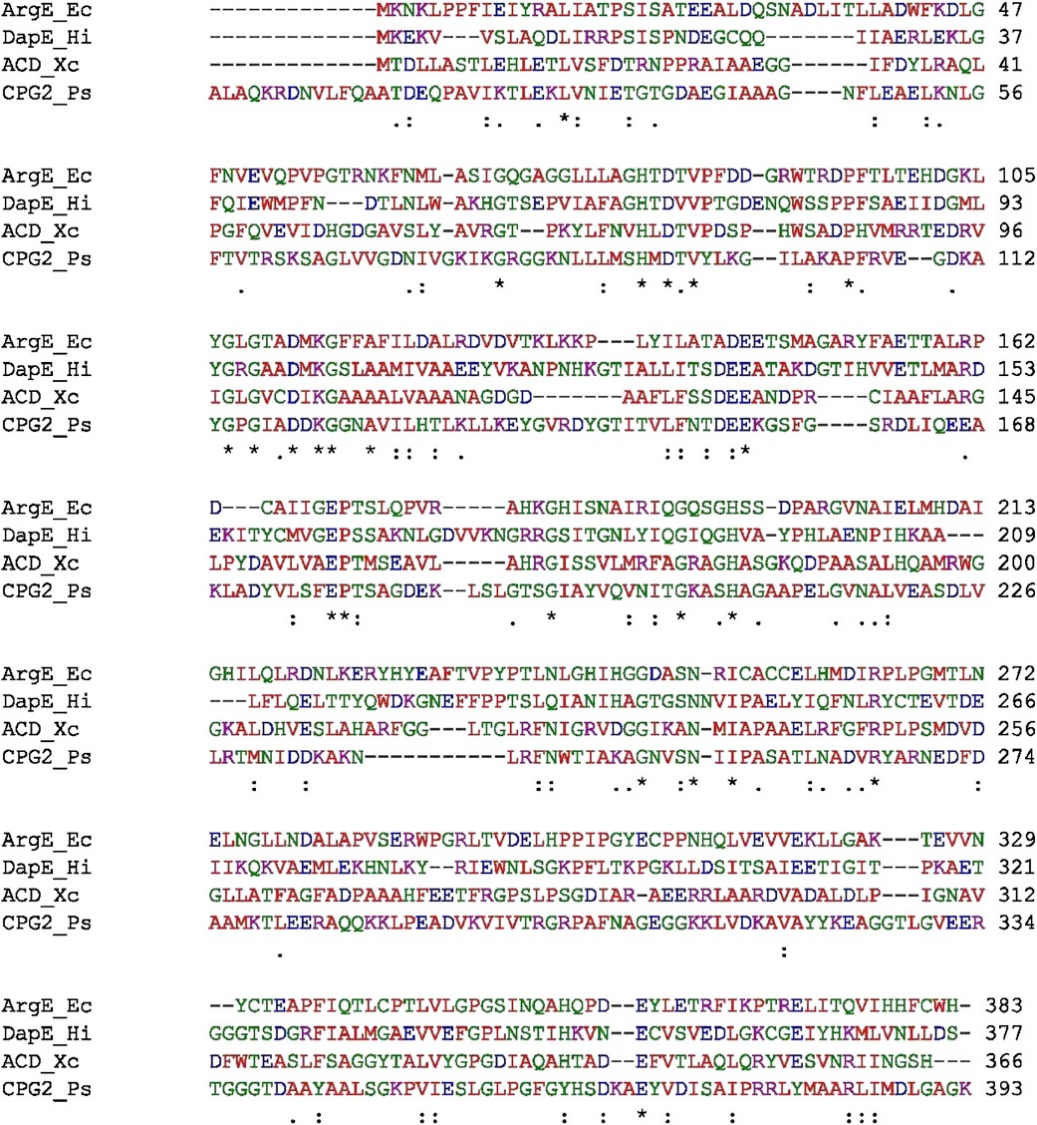
**Sequence alignment between the structurally characterized enzymes DapE from** ***H. influenzae*** **, ACD from** ***X. campestris*** **, and CPG**_**2**_ **from** ***Pseudomonas sp*** **. strain RS-16 with the ArgE from** ***E. coli*** **.**

In an effort to provide insight into the structural properties of each divalent metal ion in the active site of the ArgE form *E. coli*, we have prepared and purified the H355A, H355K, H80A, and H80K ArgE mutant enzymes. Only the H355A mutant ArgE enzyme was expressed as a soluble enzyme and, consequently, it was the only ArgE mutant investigated *via* kinetic, electronic absorption, and isothermal titration calorimentry (ITC). A three-dimensional homology model of ArgE was also generated using the X-ray crystal structure of the DapE from *Haemophilus influenzae*. Combination of these data provides verification that H355 is an active site ligand and provide new insight into the divalent metal binding properties of co-catalytic metallohydrolase active sites.

## Materials and methods

### Reagents

All chemicals used were purchased commercially and were of the highest quality available. All metal reagents were obtained from Strem Chemicals, Newburyport, MA with ≥99.999% purity. Stock solutions of Co (II) (50 mM) and Zn(II) (10 mM) were prepared by dissolving the metal chloride salts (CoCl_2_^.^6H_2_O or ZnCl_2_) in nanopure water.

### Enzyme purification

The aminopeptidase from *Aeromonas proteolytica* (AAP) was purified according to the previously published procedure (Chen et al. 1997). Protein concentrations were estimated from the absorbance at 280 nm using an extinction coefficient *ϵ*_280_ = 41,800 M^-1^cm^-1^ (Bennett and Holz 1997). Wild-type recombinant ArgE from *E. coli* was expressed and purified, as previously described, from a stock culture of *E. coli* BL21 Star™ cells (Invitrogen, Carlsbad, CA) kindly provided by Professor John Blanchard (Javid-Majd and Blanchard 2000). Pure ArgE enzyme (> 98%), as determined by SDS-PAGE gel electrophoresis, was stored at -80°C until used. The concentration of ArgE was estimated from the absorbance at 280 nm using an extinction coefficient *ϵ*_280_ = 41,250 M^-1^cm^-1^ (McGregor et al. 2005). Enzyme concentrations determined by molar absorptivity were in close agreement to that obtained using a Bio-Rad assay.

### Site-Directed Mutagenesis

The pET-27a(+) plasmid containing the *argE* gene was extracted and transformed into *E. coli* BL21 Star™ cells for expression of protein variants, which were purified in the same manner as WT ArgE. The Quick Change™ Site-Directed Mutagenesis Kit (Stratagene, La Jolla, CA, USA) and the following primers: 5’-GGC TCA ATT AAT CAG GCT XXX CAA CCT GAT GAA TAT CTG G-3’and 5’-C CAG ATA TTC ATC AGG TTG YYY AGC CTG ATT AAT TGA GCC-3’ with GCT and AAA for XXX and AGC and TTT for YYY were employed for production of the H355A and H355K variants respectively. Likewise, 5’-G CTG GCG GGG XXX ACC GAT ACG GTG CC-3’ and 5’-GG CAC CGT ATC GGT YYY CCC CGC CAG C-3’ with GCT and AAA for XXX and AGC and TTT for YYY were employed for production of H80A and H80K variants respectively. Products from these Site-Directed Mutagenesis reactions were transformed into *E. coli* XL1-Blue competent cells which were subsequently spread on LB-agar plates containing 100 μg/mL of Ampicillin. Following over-night incubation at 37°C, one of many colonies from each plate was selected for growth in 50 mL liquid LB medium, containing 100 μg/mL of Ampicillin, for ≥10 hours at 37°C and shaking at 225 rpm. Plasmids were isolated from the resultant cultures using the Qiaprep® Spin Miniprep kit (Qiagen, Valencia, CA) and incorporation of the variant gene sequence was confirmed by DNA sequencing.

### Enzymatic assay of ArgE

ArgE activity was measured by the method of Javid-Majd and Blanchard (Javid-Majd and Blanchard 2000). In this assay, the hydrolysis of a 2 mM L-NAO solution, in 50 mM Chelex-100 treated phosphate buffer at pH 7.5, was measured spectrophotometrically at 25°C as the decrease in absorbance at 214 nm (Δϵ_214_ = 103 M^-1^cm^-1^), corresponding to the cleavage of the L-NAO amide bond. Protein concentrations were determined using the theoretical value ϵ_280_ = 41,250 M^-1^ cm^-1^ (Gill and von Hippel 1989). The specific activity of purified wild-type ArgE with L-NAO was typically found to be 2,000 units per mg of enzyme. One unit was defined as the amount of enzyme that releases 1 μmole of ornithine in 60 sec at 25°C. Initial rates were fit directly to the Michaelis-Menten equation to obtain the catalytic constants *K*_m_ and *k*_cat_.

### Preparation of Apo-enzymes

Apo-enzyme samples were prepared for each purified enzyme by methods previously described in the literature (Prescott et al. 1983; Javid-Majd and Blanchard 2000). Briefly, AAP was dialyzed for 72 h at 4°C against 10 mM 1,10-phenanthroline monohydrochloride in 50 mM HEPES buffer, pH 7.5, and then exhaustively dialyzed against Chelex-treated (Chelex-100 column) 50 mM HEPES buffer, pH 7.5. (Bennett and Holz 1997). Apo-ArgE enzyme samples were prepared by adding 15 mM EDTA and incubating for 24 h at 4°C. The EDTA was removed by exhaustive dialysis against Chelex-treated (Chelex-100 column) 50 mM HEPES buffer, pH 7.5, containing 150 mM KCl at 4°C for 36 h. Metal-free buffers were prepared by passing the buffers through a Chelex-100 column. The resulting apo-enzymes were inactive, and were found to contain no detectable metals ions via Inductively Coupled Plasma Atomic Emission Spectrometry (ICP-AES).

### Spectroscopic measurements

Electronic absorption spectra were recorded on a Shimadzu UV-3101PC spectrophotometer equipped with a constant temperature holder and a Haake (Type 423) constant temperature-circulating bath. All solutions were degassed prior to performing an experiment. Electronic absorption spectra were normalized for protein concentration and the absorption due to uncomplexed Co(II) (ϵ_512_ = 6.0 M^-1^ cm^-1^) (D’souza et al. 2000).

### Isothermal Titration Calorimetry

Isothermal Titration Calorimetry (ITC) measurements were carried out on a MicroCal OMEGA ultrasensitive titration calorimeter at 25 ± 0.2°C. The divalent metal ion titrants and apo-enzyme solutions were prepared in 25 mM Chelex-100 treated HEPES buffer at pH 7.5. Stock buffer solutions were thoroughly degassed before each titration. The enzyme solution (70 μM) was placed in the calorimeter cell and stirred at 200 rpm to ensure rapid mixing. Typically, 4–6 μL of titrant was delivered over 7.6 s with 6 min. intervals between injections to allow for complete equilibration. Each titration was continued until 4.5-6 equiv. of M(II) had been added to ensure that no additional complexes were formed in excess titrant. A background titration, consisting of the identical titrant solution but only the buffer solution in the sample cell, was subtracted from each experimental titration to account for heat of dilution. Individual AAP solutions were prepared by diluting stock enzyme solutions with 50 mM Chelexed HEPES buffer (pH 7. 5), and ArgE solutions were prepared by diluting stock enzyme solutions with 25 mM Chelexed HEPES buffer (pH 7.6) containing 150 mM KCl.

### Molecular modeling

**Figure 3:**
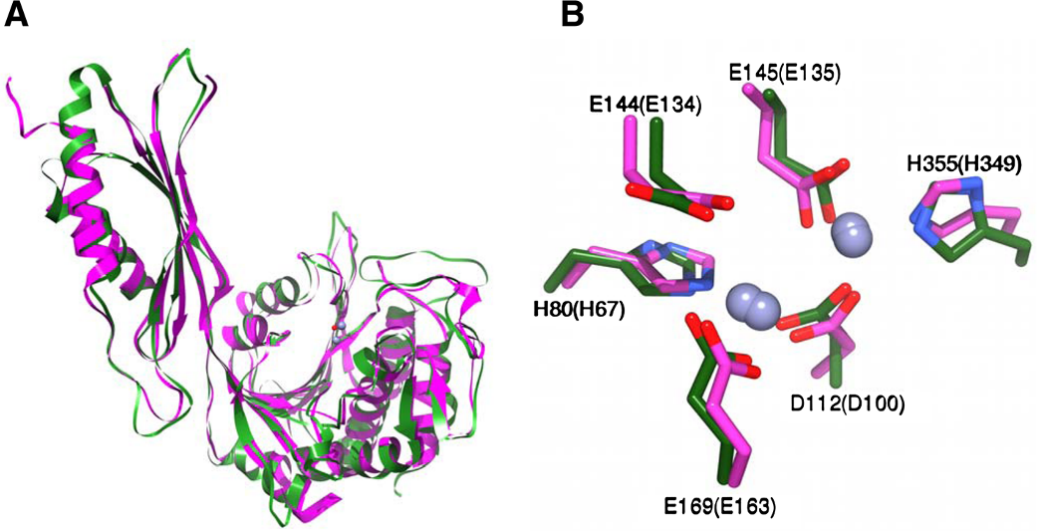
**Homology model of the [ZnZn(ArgE)] from** ***E. coli*** **based on the X-ray structure of DapE. A)** Overlay of the ArgE homology model (Green) and the X-ray crystal structure of DapE (Magenta). The two Zn(II) ions and the bridging water from ArgE are shown as spheres. **B)** Conserved active site residues for ArgE (Green) and DapE (Magenta). Nitrogen atoms are in blue, oxygen atoms are in red. The residues are labeled with single-letter amino acid codes with the labels for residues from DapE in parenthesis.

A three-dimensional homological structure of the ArgE from *E. coli* was developed using the X-ray crystal structure of the DapE from *H. influenzae* (PDB code:3IC1) as a template (Figure 3) (Rowsell et al. 1997; Eswar et al. 2003; Krissinel and Henrick 2004; Nocek et al. 2010)*.* Sequence analysis of the target and template show that these two proteins are of similar length, share good sequence identity (~24%), and exhibit no significant sequence gaps. The ModWeb-based homology-building server was used to construct a structural homology model of the ArgE from *E. coli*. Comparison of the energy minimized ArgE homology model to the X-ray crystal structure of DapE, using the MathMaker in UCSF Chimera, reveals that the ArgE homology model displays the typical dimerization and catalytic domains of DapE with a Needleman-Wunsch (Needleman and Wunsch 1970) score of 490.6 and an average RMSD of 0.974 Å for the core atom pairs (Figure 3).

## Results and discussion

### Protein production

Of the four ArgE mutant plasmids produced (H355A, H355K, H80A and H80K) and transformed into *E. coli* BL21 Star™ cells, only ArgE H355A was produced as a soluble enzyme*.* SDS-PAGE analyses of protein from *E. coli* cultures hosting plasmids containing the other three ArgE variants indicated that the enzymes were produced in abundance, but in an insoluble form. Various attempts were made to refold inclusion bodies of the three insoluble ArgE variants, but none proved fruitful.

### Kinetic studies on ArgE H355A

Based on ICP-AES data, the as-purified H355A ArgE mutant enzyme contains no Zn(II) ions. These data imply that the remaining metal binding site in ArgE has a much higher *K*_d_ value and is unable to bind Zn(II) in the absence of saturating conditions, similar to DapE, and consistent with weak cooperative binding as suggested by EXAFS (Tao et al. 2012; Gillner et al. 2009). The hydrolysis of L-NAO by H355A was monitored in the presence of three equivalents of Zn(II) or Co(II), after a one-hour incubation period, at pH 7.5 (Figure 1). Interestingly, no enzymatic activity was detected for H355A ArgE in the presence of three equivalents of Zn(II). The lack of measurable activity for the H355A mutant enzyme may be the result of the reaction conditions used. Since the amide bond cleavage of L-NAO is monitored at 214 nm, enzyme concentrations must be < 10 nM so as not to overwhelm the amide bond absorption of L-NAO with background protein absorption. The severely reduced/eliminated enzymatic activity is consistent with the impaired ability of this variant to bind Zn (II). This further substantiates the hypothesis that this point mutation has knocked out a key metal ligand.

Further verification of this hypothesis comes from examining the hydrolysis of L-NAO by the Co (II)-loaded H355A variant. The WT Co (II)-loaded ArgE enzyme is 2.4-times more active than the WT Zn (II)-loaded form. For Co (II)-loaded H355A ArgE, a *k*_cat_ value of 10 ± 1 s^-1^ was obtained in the presence of three equivalents of Co(II) using L-NAO as the substrate (*K*_m_ = 0.5 ± 0.1 mM). Comparison of the kinetic parameters for the Co(II)-loaded WT and H355A ArgE enzymes indicate that the decrease in activity of H355A is due to a ~380-fold decrease in *k*_cat_. Interestingly, the *K*_m_ value determined for the Co (II)-loaded H355A mutant enzyme is smaller than Co(II)-loaded WT ArgE by ~2-fold. Calculation of the catalytic efficiency (*k*_cat_/ *K*_m_) for the Co(II)-loaded H355A mutant enzyme results in a value ~160-fold less than the Co(II)-loaded WT ArgE. The histidine to alanine mutation at this position, while having some impact on substrate binding, has primarily affected a key aspect of the active site of the enzyme (*i.e*. metal cofactor binding).

### Electronic absorption spectra of Co(II)-loaded ArgE

**Figure 4:**
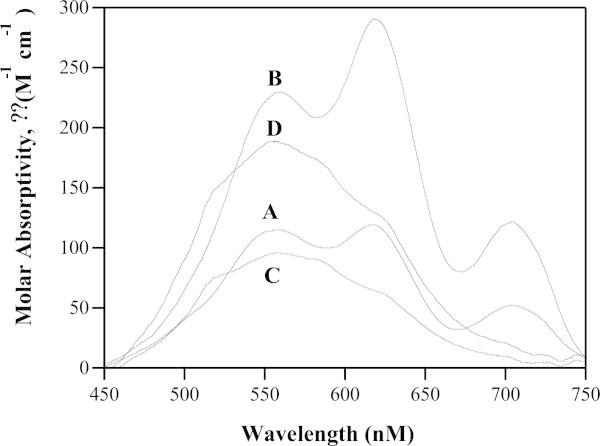
**UV–vis absorption spectra of 1 mM samples of (A) WT ArgE in the presence of 1 equivalent of Co(II), (B) WT ArgE in the presence of 2 equivalents of Co(II), (C) H355A ArgE in the presence of 1 equivalent of Co(II), and (D) H355A ArgE in the presence of 2 equivalents of Co(II).** (Reaction conditions: 25°C in 50 mM HEPES buffer, pH 7.5, and 150 mM KCl).

Insight into whether H355 acts as an active site Zn(II) ligand was also investigated by examining the electronic absorption spectra of the Co(II)-substituted forms of the enzyme (Figure 4). The addition of one equivalent of Co(II) to apo-H355A ArgE (in Chelex-100 treated 50 mM HEPES buffer at pH 7.5) results in a broad absorption band with maxima at 560 nm (ϵ_560_ = ~95 M^-1^ cm^-1^) along with shoulders at 515 nm (ϵ_515_ = ~75 M^-1^ cm^-1^) and 620 nm (ϵ_515_ = ~55 M^-1^ cm^-1^) suggesting that the Co(II) ion bound to H355A ArgE resides in a penta-coordinate geometry (Bertini and Luchinat 1984; Horrocks et al. 1980). The addition of a second equivalent of Co(II) to H355A ArgE did not alter the overall shape of the observed absorption spectrum but did approximately double the molar absorptivity, consistent with a coordination number of five. In contrast, the WT ArgE from *E. coli* in the presence of one equivalent of Co(II) exhibited three distinct peaks at 560, 619, and 705 nm with *ϵ* values of *ϵ*_560_ = 114 M^-1^cm^-1^, *ϵ*_619_ = 119 M^-1^cm^-1^, and *ϵ*_705_ = 52 M^-1^cm^-1^, respectively (McGregor et al. 2005). The addition of a second equivalent of Co(II) increased the intensity of each of the observed absorption bands, providing *ϵ* values of *ϵ*_560_ = 229 M^-1^cm^-1^, *ϵ*_619_ = 290 M^-1^cm^-1^, and *ϵ*_705_ = 121 M^-1^cm^-1^, respectively (Figure 4). Thus, the UV–vis spectrum of H355A ArgE is significantly different from WT ArgE in the presence of one and two equivalents of Co(II) (Figure 4) and the loss of distinct d-d transitions in the UV–vis spectrum of H355A is likely the result of a more flexible distorted five-coordinate geometry with water molecules occupying non-protein coordination sites. Taken together, these data are consistent with the loss of an active site histidine ligand.

**Figure 5:**
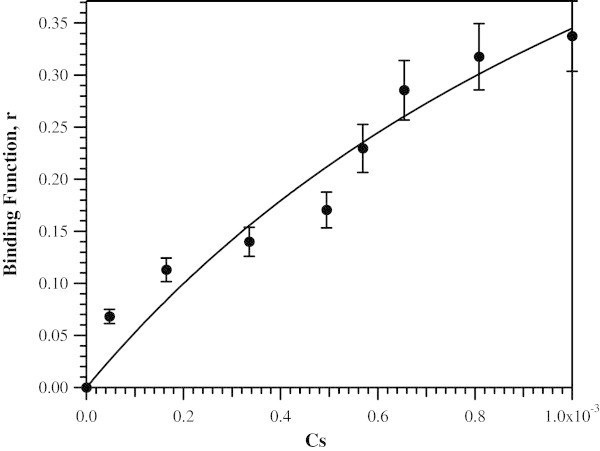
**Plot of Δϵ**_**560**_ **vs. the concentration of free Co(II) ions in solution for Co(II) titration into apo-H355A ArgE (50 mM KH**_**2**_ **PO**_**4**_ **buffer, pH 7.5).**

The altered ability of the H355A ArgE to bind Co(II) was also investigated by titrating Co(II) into apo-H355A ArgE samples and monitoring the molar absorptivity of the λ_max_ for the Co(II) *d*-*d* bands, which allowed the dissociation constant (*K*_d_) for this divalent metal binding site to be obtained by fitting these titration data to equation 1 (Winzor and Sawyer 1995):
1

where *p* is the number of sites for which interaction with Co(II) is governed by the intrinsic dissociation constant *K*_d_, C_s_ is the free metal concentration and, *r* is the binding function calculated by conversion of the fractional saturation (*f*_a_) (Winzor and Sawyer 1995). Values for *K*_d_ and *p* were obtained by fitting these data *via* an iterative process that allowed *K*_d_ and *p* to vary (Figure 5). The best fits obtained for Co(II) binding to the H355A ArgE provided a *p* value of 0.9 and a *K*_d_ value of 1.6 ± 0.3 μM, which is 4-fold larger than that observed for WT ArgE (*K*_d_ = 0.4 μM) (McGregor et al. 2005) consistent with a loss of an active site ligand. Since alteration of H355 will greatly perturb and potentially alter the ability of metal binding, this *K*_d_ value likely corresponds to a single metal binding event. Moreover, the *K*_d_ value obtained for Co(II) binding to H355A is ~4 times stronger than that obtained for Zn(II) binding to WT ArgE. This difference is typically related to Zn(II)’s preference for four or five-coordinate geometries *vs*. Co(II)’s preference for five or six-coordinate structures (Holz 2002). These data, taken together with the lack of observed activity for H355A ArgE in the presence of Zn(II), suggest that H355 is in fact an active site ligand.

### Isothermal Titration Calorimetry

**Figure 6:**
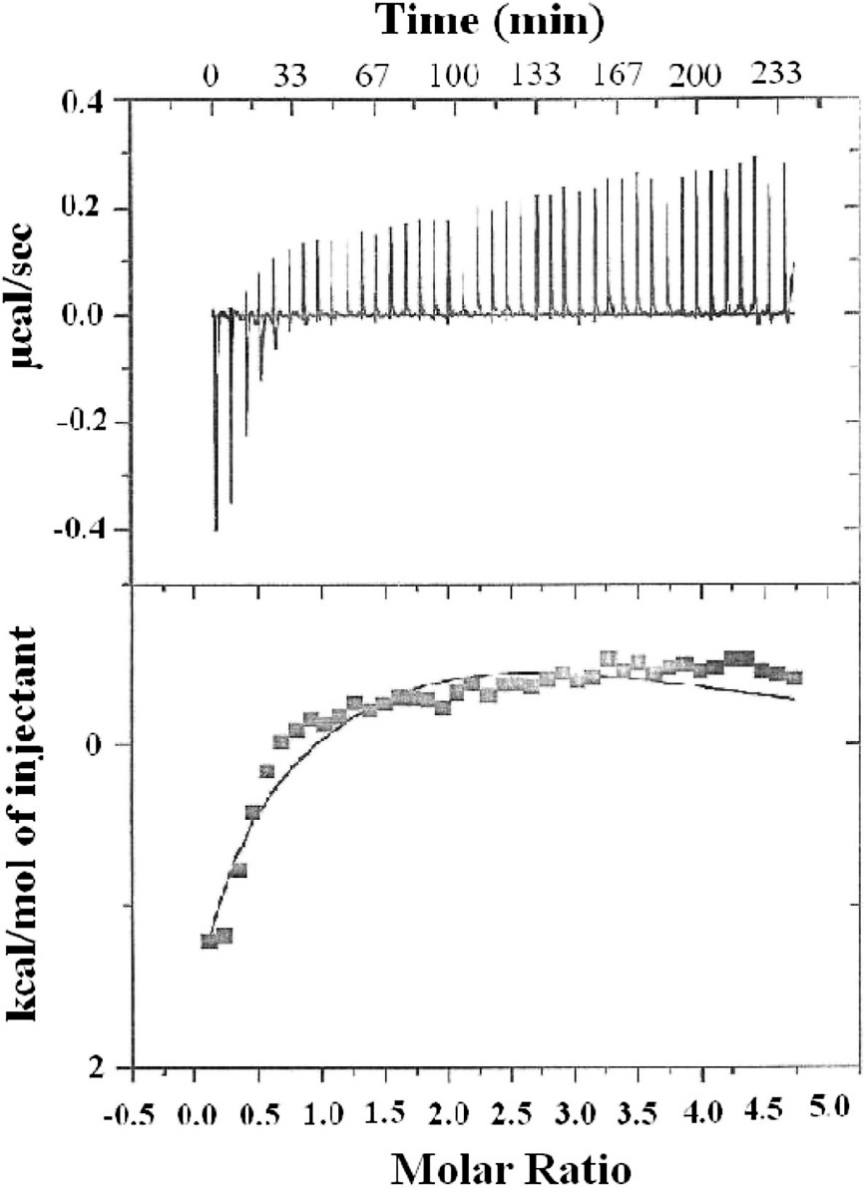
**(Top) ITC titration data for a 70 μM solution of apo- H355A ArgE with a 2.5 mM solution of Zn(II).** (Bottom) Fit of the ITC data for H355A ArgE after subtracting the heat of dilution. (Reaction conditions: 25°C in 50 mM HEPES buffer, pH 7.5, and 150 mM KCl).

Additional insight into whether H355 is a ligand in the active site of ArgE and which metal binding site it resides in, were gleaned by obtaining *K*_d_ values and thermodynamic profiles using ITC (Figure 6). These data were analyzed with a one, two, or three-site binding model after subtraction of the background heat of dilution, *via* an interactive process using the Windows-based Origin software package supplied by MicroCal. This software package uses a nonlinear least-squares algorithm, which allows the concentrations of the titrant and the sample to be fit to the heat-flow-per-injection to an equilibrium binding equation. The *K*_a_ value, enzyme-metal stoichiometry (n), and the change in enthalpy (*ΔH*^o^) were allowed to vary during the fitting process. The equilibrium binding constant, *K*_a_, and the enthalpy change Δ*H*, were used to calculate Δ*G* and Δ*S* using the Gibbs free energy relationship (equation 2):
2

where R = 1.9872 cal mol^-1^ K^-1^. The relationship between *K*_a_ and *K*_d_ is defined as:
3

For the H355A ArgE mutant, the best fits provided *n* values of 2 for two non-interacting sites with *K*_d_ values of 39 and 330 μM (Figure 6; Table 1). The ITC data do not suggest cooperative binding whereas the EXAFS data do; however, weak cooperative binding is typically undetectable *via* ITC. The Δ*H*^o^ value for the first Zn (II) binding event is endothermic with a large positive entropy consistent with the loss of water ligands from the Zn(II)-hexaauqo complex upon Zn(II) binding to ArgE. On the other hand, the second Zn(II) binding event is exothermic with an entropy change near zero (Table 2).

**Table 1:** **Metal dissociation constants of ArgE H355A and WT ArgE determined by ITC and UV–vis absorption titration**

Enzyme	Metal	Method	***K***_d1_ (μM)	***K***_d2_ (μM)
WT ArgE^a^	Zn(II)	ITC	2.7	51
H355A ArgE	Zn(II)	ITC	39	330
WT ArgE^a^	Co(II)	ITC	0.4	153
H355A ArgE	Co(II)	UV–vis Titration	1.6	ND^b^

^a^Previously reported (ref. (McGregor et al. 2005)).

^b^None detected.

**Table 2:** **Thermodynamic binding parameters for Zn(II) binding to AAP, WT ArgE and H355A ArgE (ΔH (kcal/mol), (ΔG cal/mol·K) and (ΔS cal/mol·K))**

Parameter	AAP	WT ArgE ^a^	H355A ArgE
**Δ** ***H***_***1***_	2.1	4.6	6.0
**Δ** ***H***_***2***_	1.1	-2.7	-3.7
**Δ** ***G***_***1***_	-11.0	-5.9	-6.0
**Δ** ***G***_***2***_	-7.5	-7.6	-4.7
**Δ** ***S***_***1***_	43	35	40
**Δ** ***S***_***2***_	29	16	3.5

^a^Previously reported (ref. (McGregor et al. 2005)).

**Figure 7:**
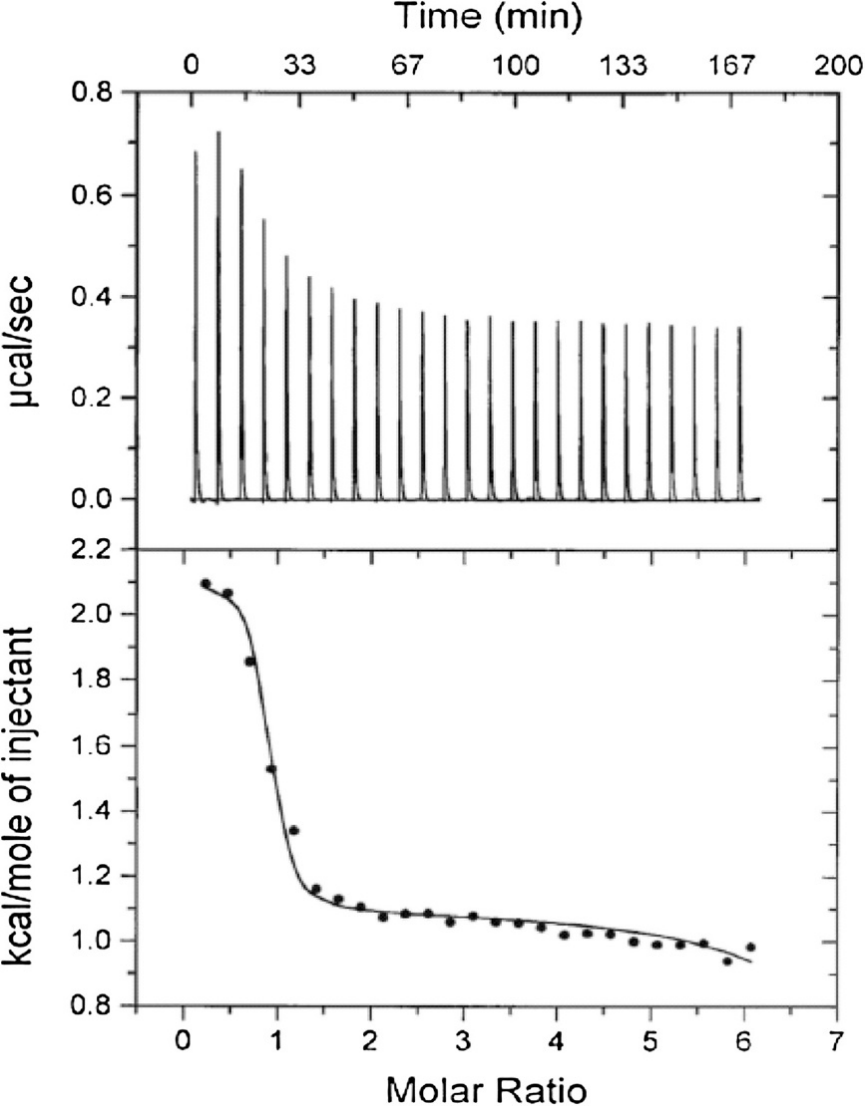
**(Top) ITC titration data for 30 μM solution of AAP with a 2.5 mM Zn(II) solution. (Bottom) Fit of the ITC data for AAP after subtraction of the heat of dilution.** (Reaction conditions: 25°C in 50 mM HEPES buffer, pH 7.5).

Comparison of these data with previously reported ITC data for Zn(II) binding to WT ArgE and DapE provide insight into which metal binding site is being populated first in the H355A ArgE mutant enzyme (McGregor et al. 2005; Davis et al. 2006). Both ArgE and DapE bind two Zn(II) ions in non-interactive binding sites with *K*_d_ values for the first Zn(II) binding event for ArgE and DapE being 2.7 and 4.4 μM, respectively, whereas the observed *K*_d_ values for the second metal binding event in ArgE and DapE are 51 and 13.6 μM, respectively. The Δ*H*^o^ values observed for the first Zn(II) binding event in both ArgE and DapE are endothermic while the second is exothermic (Table 2). While *K*_d_ for AAP has been reported previously by one of us (Bzymek et al. 2005), we report here a high quality ITC data set for Zn(II) binding to AAP, which provided *K*_d_ values for the first and second metal binding event of 0.008 and 0.4 μM, respectively (Figure 7). The energy change observed for both AAP binding sites indicates an endothermic-type reaction with the second metal binding event providing a more negative Δ*H*^o^ value than the first. Taken together, the thermodynamic data obtained for H335A are similar to those observed for the first binding events in WT ArgE, DapE, and AAP, suggesting that the H335 residue resides in the second metal binding site similar to that observed for DapE (Gillner et al. 2009; Nocek et al. 2010). The weaker binding affinity for the first Zn(II) binding event in H355A ArgE vs. WT ArgE is likely the result of weak cooperative binding, which would be lost upon mutation of H355.

### Modeling of the ArgE structure

**Figure 8:**
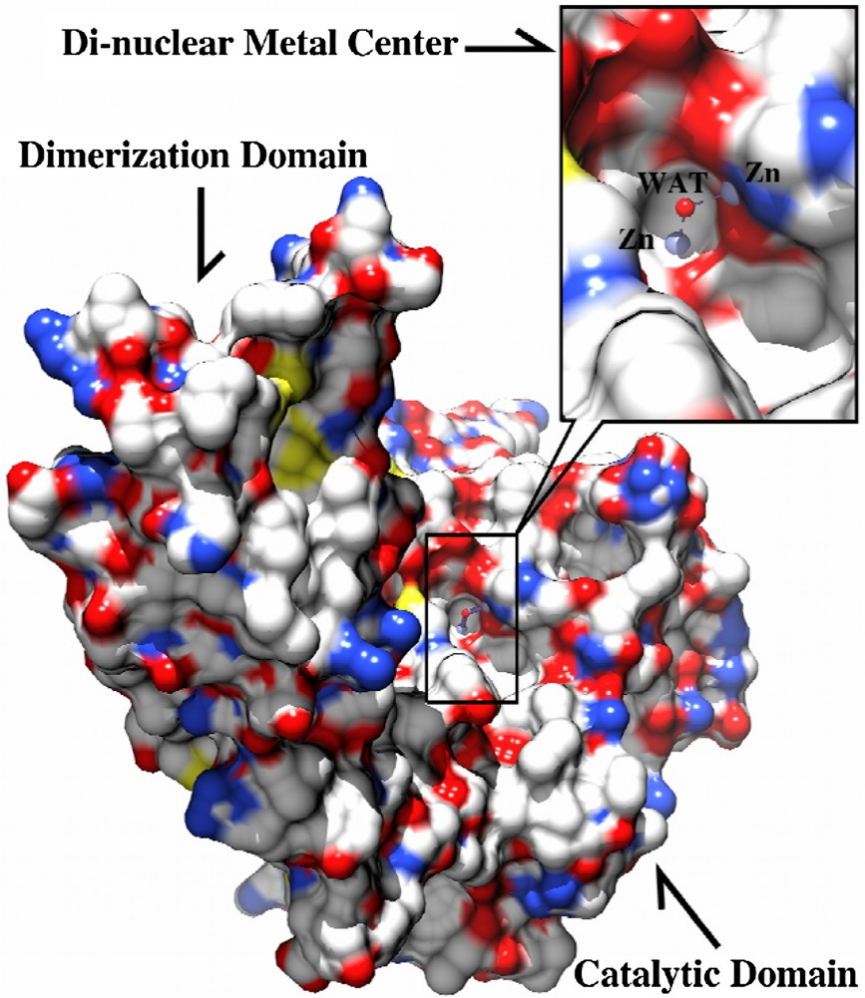
**Surface rendering of the [ZnZn(ArgE)] molecule where the black box indicates the position of the di-nuclear metal center.** Insert: Close-up of the dinuclear Zn(II) cluster showing that the active site is located in a long cleft across the catalytic domain. Zn(II) ions are shown in light blue spheres and the bridging water molecule is shown as a red sphere and labeled as “WAT”.

Since no three-dimensional X-ray crystal structure has been reported for the ArgE form *E. coli*, a three-dimensional homological structure was developed using the X-ray crystal structure of the DapE from *H. influenzae* (PDB code:3IC1) as a template (Figure 3) (Rowsell et al. 1997; Eswar et al. 2003; Krissinel and Henrick 2004; Nocek et al. 2010)*.* The catalytic domain of ArgE contains the dinuclear Zn(II) site with a Zn-Zn distance of ~3.3 Å and within 4.0 Å of the Zn(II) binding sites of ArgE, and all of the amino acid residues are nearly identical to those observed in DapE confirming the assignment of H80 and H355 as active site ligands in ArgE (Figure 3) (Rowsell et al. 1997; Eswar et al. 2003; Krissinel and Henrick 2004). The active site in ArgE is located in a long and open cleft of the catalytic domain; the cleft is parallel to and in the vicinity of the “hinge” region between the catalytic domain and the dimerization domain (Figure 8). With such a location, conformational changes upon metal and/or substrate binding are likely and have been observed for DapE (Nocek et al. 2010). Both Zn(II) ions are solvent accessible and have four protein centered ligands including H80 and H355 as well as two terminal glutamate residues (E145 and E160) and a bridging aspartate residue (D122). A bridging water provides the fifth ligand for both metal ions, which forms a hydrogen bond to a second water molecule that is in turn hydrogen bonded to E144, a candidate for the general acid/base during catalysis (Davis et al. 2006). Therefore, our data supports the hypothesis that H355 is an active site Zn (II) ligand, and as this residue corresponds to H349 in DapE, which has been shown to be the second metal binding site, it is likely that H355 resides in the second metal binding site of ArgE.

### Summary

The kinetic, spectroscopic and thermodynamic data presented herein provide evidence that H355 is intimately involved in catalysis and serves as a metal binding ligand. While these data do not conclusively indicate which metal binding site H355 resides in, they are consistent with H355 residing in the second metal binding site, similar to DapE. Therefore, the ArgE from *E. coli* forms a (μ-aquo)(μ-carboxylato)dizinc(II) core where H80 and H355 function as active site metal ion ligands similar to DapE, AAP, and CPG_2_ and consistent with EXAFS data (Tao et al. 2012). While the data reported here provide a structural starting point for elucidating the catalytic mechanism of ArgE, which may lead to the design and synthesis of small molecule inhibitors that specifically target ArgE, an X-ray crystal structure of the ArgE from *E. coli* with both one and two metal ions bound is desperately needed.

## Abbreviations

ArgE: *argE*-encoded *N*-acetyl-L-ornithine deacetylase
DapE: *dapE*-encoded *N*-succinyl-L,L-diaminopimelic acid desuccinylase
AAP: aminopeptidase from *Vibrio proteolyticus (Aeromonas proteolytica)*
ACD: *N*-acetyl-L-citrulline deacetylase from *Xanthomonas campestris*
CPG2: carboxypeptidase from *Pseudomonas sp.* strain-RS-16
L-NAO: *N*^α^-acetyl-L-ornithine
EDTA: Ethylenediaminetetraacetic acid
HEPES: 4-(2-hydroxyethyl)-1-piperazineethanesulfonic acid
Tris: tris(hydroxymethyl)aminomethane
Tricine: *N*-tris[hydroxymethyl]methylglycine
SDS-PAGE: Sodium Dodecyl Sulfate-Polyacrylamide Gel Electrophoresis
ITC: Isothermal Titration Calorimetry
ICP-AES: Inductively Coupled Plasma Atomic Emission Spectrometry.
